# Draft Genome Sequence of the Lignocellulose Decomposer *Thermobifida fusca* Strain TM51

**DOI:** 10.1128/genomeA.00482-13

**Published:** 2013-07-11

**Authors:** Ákos Tóth, Terézia Barna, István Nagy, Balázs Horváth, István Nagy, András Táncsics, Balázs Kriszt, Erzsébet Baka, Csaba Fekete, József Kukolya

**Affiliations:** Department of Environmental Protection & Environmental Safety, Szent István University, Gödöllő, Hungarya; Department of Genetics and Molecular Biology, University of Debrecen, Debrecen, Hungaryb; Institute of Biochemistry, Biological Research Centre of the Hungarian Academy of Sciences, Szeged, Hungaryc; Department of Structural Biology, Max Planck Institute of Biochemistry, Martinsried, Germanyd; Regional University Center of Excellence in Environment, Industry, Szent István University, Gödöllő, Hungarye; Szentágothai János Research Center, Pécs, Hungaryf; Department of Microbiology, Central Environmental and Food Science Research Institute, Budapest, Hungaryg

## Abstract

Here, we present the complete genome sequence of *Thermobifida fusca* strain TM51, which was isolated from the hot upper layer of a compost pile in Hungary. *T. fusca* TM51 is a thermotolerant, aerobic actinomycete with outstanding lignocellulose-decomposing activity.

## GENOME ANNOUNCEMENT

Thermobifidas are aerobic actinomycetes belonging to *Nocardiopsaceae*. The genus consists of four species, namely *T. fusca*, *T. cellulosilytica*, *T. alba*, and *T. halotolerans* (1).

The Yellowstone hot spring isolate *T. fusca* strain YX is by far the most characterized species from this taxon; its genome was published in 2007 (2). D. B. Wilson’s pioneering works elucidated the cellulase system of *T. fusca* YX, and despite the lack of genetic tools this strain became the model organism of thermotolerant aerobic microbial cellulose decomposers (3). In addition to cellulases it produces a series of thermostable hemicellulases, including xylanase, mannanase, xyloglucanase, and amylase enzymes with a high level of industrial importance (4–8).

Here we report the genome sequence of *T. fusca* strain TM51, which was isolated from the hot upper layer of a horse manure compost pile. While the source of isolation was a piece of decomposed straw fiber at 80°C, the isolated strain can grow only up to 69°C (10). When TM51 grows on a crystalline cellulose (MN300)-containing agar plate, the colonies appear with a white color due to the billions of spores, which are formed on the tip of the dichotomically branched aerial mycelium revealed by a scanning electron microscope (9). Strain TM51 is not only an outstanding cellulose degrader; it can also rapidly decompose xylan and mannan substrates. During the last decade an endoglucanase (Cel5B), a mannosidase (Man2A), and a beta-xylosidase (Xyl43A) were cloned and biochemically characterized from this strain (10–12).

Genome sequencing of *T. fusca* strain TM51 was performed by the SOLiD (Life Technologies) mate-paired sequencing technology. We have generated 6,352,623 mate-paired (2-by-25-bp) reads, which yielded >80-fold coverage. Assembly was performed using the Genomics Workbench 4.9 (CLC Bio). Automatic annotation of the genome was performed by the NCBI Prokaryotic Genomes Automatic Annotation Pipeline (PGAAP) (http://www.ncbi.nlm.nih.gov/genome/annotation_prok/). We have assembled the genome of *T. fusca* strain TM51 into 88 contigs with a reference length of 3,599,272 bp, 3,080 putative coding sequences, 52 tRNAs, and 12 rRNAs.

The analysis of the annotated genome revealed the existence of 39 putative glycoside hydrolases belonging to 23 different glycoside hydrolase families (13). From this enzyme pool 18 enzymes, mainly cellulases, xylanases, and mannanases, have been described. GH13 is the largest glycosyl hydrolase family in TM51, with six enzymes predicted to exhibit mainly dextran- and starch-degrading functions. By the pairwise comparison of the glycosyl hydrolases of *T. fusca* YX and TM51 origin, we found 85 single nucleotide polymorphisms (SNPs) and 28 amino acid alterations between them. Members of GH5 were found to be the most conservative group because all of them (endoglucanase Cel5A and Cel5B and endomannanse Man5A) showed 100% nucleic acid homology.

Thermobifidas may become important industrial strains due to their thermostable and robust polysaccharide-degrading enzymes (14, 15). Hence, *T. fusca* strain TM51 may play a key role in lignocellulose-based ethanol-producing projects and prebiotics production and as a source of hydrolases serving as feed additives.

### Nucleotide sequence accession numbers.

This whole-genome shotgun project has been deposited at DDBJ/EMBL/GenBank under the accession number AOSG00000000. The version described in this paper is version AOSG01000000.
